# Paternal psychosocial work conditions and mental health outcomes: A case-control study

**DOI:** 10.1186/1471-2458-8-104

**Published:** 2008-03-31

**Authors:** Stefania Maggi, Aleck Ostry, James Tansey, James Dunn, Ruth Hershler, Lisa Chen, Clyde Hertzman

**Affiliations:** 1 Department of Psychology and Institute of Interdisciplinary Studies, Carleton University, Colonel By Drive, Ottawa, Canada; 2 Department of Geography, University of Victoria, Finnerty Road, Victoria, Canada; 3 Department of Health Care and Epidemiology, University of British Columbia, Fairview Avenue, Vancouver, Canada; 4 Department of Geography, University of Toronto, St. George Street, Toronto, Canada

## Abstract

**Background:**

The role of social and family environments in the development of mental health problems among children and youth has been widely investigated. However, the degree to which parental working conditions may impact on developmental psychopathology has not been thoroughly studied.

**Methods:**

We conducted a case-control study of several mental health outcomes of 19,833 children of sawmill workers and their association with parental work stress, parental socio-demographic characteristics, and paternal mental health.

**Results:**

Multivariate analysis conducted with four distinct age groups (children, adolescents, young adults, and adults) revealed that anxiety based and depressive disorders were associated with paternal work stress in all age groups and that work stress was more strongly associated with alcohol and drug related disorders in adulthood than it was in adolescence and young adulthood.

**Conclusion:**

This study provides support to the tenet that being exposed to paternal work stress during childhood can have long lasting effects on the mental health of individuals.

## Background

The etiology and contributing factors of mental health problems are complex and multifaceted. Researchers have investigated a broad range of factors that may contribute to the development of mental health problems such as a family history of mental health problems and socio-demographic factors (e.g., ethnicity and marital status).

There is growing evidence that ethnicity and other parental characteristics may impact on child mental health outcomes through socialization practices (for a review see Hughes et al. [1]). For example, increasingly research is documenting that positive ethnic identity among minority children and adolescents functions as a protective factor for mental health and school outcomes, and it has been hypothesized that such protective effect is a function of socialization processes occurring between parents and children [2-4].

While research on how specific ethnic socialization processes impact on child and adolescent outcomes is still needed, the role of the social and family environments in the development of mental health problems among the non-minority children and youth has been widely investigated. Theoretical models of developmental psychopathology have highlighted the importance of environmental stressors in the etiology of different mental health outcomes [5-8]. Some of these stressors include acute traumatic events, chronic strain and adversity, and accumulation of stressful life events and daily challenges [7]. Some of the most notable environmental stressors that have been found to profoundly impact children and youth mental health are exposure to neighbourhood violence [9]; parental chronic illness [10-13]; and poverty and economic hardship [12]. In addition, temporary or prolonged parental unemployment may add further stress in the form of increased parental alcohol intake, home violence and child abuse [13].

Research on the impact of unemployment on children's development has identified changes in parenting and marital relationships as the primary factors linked to children's socio-emotional functioning [14-16]. Several studies originating in the Great Depression of the 1930s indicated that fathers were more susceptible than mothers of emotional instability as a consequence to major income family losses [17,18]. Specifically, fathers tended to have increased anger and be more punitive in disciplining their children [15,19,20].

Parental socioeconomic difficulties due to prolonged unemployment or loss of income are also associated with increased addictive behaviours such as alcohol dependence and alcoholism, and family violence [13] which in turn increase the risk of children living in such family environments to develop poor mental and physical health in adulthood [21-23]. Several studies postulated that the way in which socioeconomic hardship affects parenting styles, especially in fathers, functions as an important determinant of children's mental health outcomes and school related problems [24-28].

As the effect of socioeconomic hardship and unemployment can be mediated through changes in parenting styles, adverse psychosocial work conditions experienced by employed fathers can also play a critical role in children's outcomes.

In addition to the specific type of employment, the degree to which fathers experience stress at the workplace can also contribute significantly to the quality of the family environment which is acknowledged as a critical determinant of children's outcomes.

Two studies have been conducted to date on the impact of psychosocial work conditions of employed fathers on children's outcomes. Steward and Barling [25] conducted a study with 189 grade 4 and 5 Canadian students and found that paternal psychosocial work conditions were associated with parenting behaviours and children's behaviour. Ostry and colleagues [29] conducted a study with 19,833 Canadian children of sawmill workers and found significant associations between suicidal behaviours and several psychosocial work conditions of fathers when their children were young or in their early adolescence.

These findings, in conjunction with epidemiological trends indicating that the onset of many mental health disorders occurs in childhood and adolescence [30-35], emphasize the importance of understanding how exposure to paternal psychosocial work conditions in childhood influence mental health outcomes throughout the lifespan.

This study focuses on the investigation of the components of the job strain model [36], also known as the demand/control model, as determinants of mental health outcomes across four broad age groups spanning from childhood to adulthood. The demand/control model postulates that job strain occurs when workers are overloaded psychologically and at the same time they have no control over their work environment. This combination of high psychological demand and low control is hypothesized to increase the risk of stress related illnesses amongst the workers [37].

Here we hypothesize that paternal psychosocial work conditions play an important role in the onset of mental health problems and that, consistent with the literature on developmental psychopathology, paternal mental health and socio-demographic characteristics can be significant determinants of mental health condition from childhood to adulthood.

Therefore, the present study investigates the impact of paternal psychosocial work conditions on a broad range of mental health conditions in a cohort of children whose fathers were employed in a selected group of sawmills in British Columbia, Canada. In recognition of the importance of addressing mental health outcomes from a developmental perspective, we investigate the onset of mental health conditions in four different age groups: childhood and early adolescence, adolescence, young adulthood, and adulthood. Because the influence of stressors on mental health can change considerably from childhood to adolescence and throughout adulthood [38-40] conducting separate analysis for these four groups has allowed us to investigate the specific effects of paternal psychosocial work conditions on mental health outcomes from a life-course perspective.

## Methods

This study is based on a cohort of male sawmill workers (N = 28,794) for whom data on employment history, job mobility, and physical and psychosocial work conditions were obtained. The original cohort of sawmill workers was gathered in the mid 1990s in order to conduct an occupational study on the effects of chlorophenol anti-sapstain exposure among British Columbian sawmill workers. Fourteen sawmills located in British Columbia were identified and personnel records for workers who had worked in one of these mills for at least one year in the period between1950 and 1998 were accessed and reviewed. Personal identifying information for eligible workers and complete job history records were abstracted from personnel records (see Hertzman et al. [41] for a complete description of methods used to assemble the sawmill workers' cohort).

Using birth files from the British Columbia provincial vital statistics registry and BC Linked Health Data Base we identified the children born between 1952 and 2000 to the cohort of sawmill workers. Through these two linkages 37,827 children of sawmill workers were identified, forming an offspring cohort. This study focuses on the developmental psychopathology among the offspring cohort and its association with paternal psychosocial work conditions.

### Study participants

The cohort of adult sawmill workers (i.e., fathers) was linked to the British Columbia birth file in order to identify all of the children of these workers born in British Columbia between 1952 and 2000. There were 37, 827 children in the cohort. Ages of the children in the cohort ranged from less than one year old to 49 years old in 2000. In order to meet the eligibility criteria for this study, the fathers must have worked at least one year in one of the study sawmills while their children were between the age of 0 and 16 years. This criterion was set to ensure that the psychosocial work conditions had been measured for the period during childhood and early adolescence. A total of 19,833 children of sawmill workers satisfied the eligibility criterion for inclusion.

In the International Classification of Diseases, 9th Revision (ICD9), children and adults are defined based on the age cut-off of 14. There are different diagnostic codes for the paediatric (14 years and younger) and adult (15 and older) populations. However, it is arguably reductionist, and some may say erroneous, to consider all individuals 15 years and older as adults, given the well documented developmental differences between adolescence and adulthood. Therefore, we used general knowledge of broad differentiations between developmental stages to classify 15 years old and older individuals in three age groups: adolescents, young adults and adults. Thus, the four groups identified were: children who had been diagnosed with mental health problems for the first time (i.e., onset) by a health professional at 14 years of age or younger (children and early adolescent group); adolescents whose onset of a mental health problem had been diagnosed between 15 and 19 years of age (adolescent group); young adults whose onset of a mental health problem had been diagnosed between 20 and 30 years of age (young adult group); and adults whose onset of a mental health problem had been diagnosed between 31 and 49 years of age (adult group). Table 1 indicates the number of cases identified for each of the mental health conditions analyzed in the present study among the four age groups.

**Table 1:** Number of cases per mental health diagnosis

*Children *(N = 19,833)		Cases	Controls	Total
ICD9 300	Neurotic Disorders	119	357	476
ICD9 301	Personality Disorder	32	96	128*
ICD9 308	Acute Reaction to Stress	48	144	192
ICD9 309	Adjustment Reaction	118	354	472
ICD9 311	Depression	187	561	748

*Adolescents*		Cases	Controls	Total

ICD9 300	Neurotic Disorders	344	1032	1376
ICD9 301	Personality Disorder	81	243	324
ICD9 308	Acute Reaction to Stress	181	543	724
ICD9 309	Adjustment Reaction	187	561	748
ICD9 311	Depression	643	1929	2572
ICD9 303	Alcohol Dependence	36	108	144
ICD9 304	Drug Dependence	57	171	228
ICD9 305	Non-Dependent Drug Abuse	74	222	296

*Young Adults*		Cases	Controls	Total

ICD9 300	Neurotic Disorders	1058	3174	4232
ICD9 301	Personality Disorder	163	489	652
ICD9 308	Acute Reaction to Stress	651	1953	2604
ICD9 309	Adjustment Reaction	404	1212	1616
ICD9 311	Depression	1682	5046	6728
ICD9 303	Alcohol Dependence	147	441	588
ICD9 304	Drug Dependence	243	726	969
ICD9 305	Non-Dependent Drug Abuse	195	585	780

*Adults*		Cases	Controls	Total

ICD9 300	Neurotic Disorders	725	2175	2900
ICD9 301	Personality Disorder	151	453	604
ICD9 308	Acute Reaction to Stress	468	1404	1872
ICD9 309	Adjustment Reaction	324	972	1296
ICD9 311	Depression	1118	3354	4472
ICD9 303	Alcohol Dependence	125	375	500
ICD9 304	Drug Dependence	173	519	692
ICD9 305	Non-Dependent Drug Abuse	117	351	468

* These diagnostic codes were eliminated from the analysis because the ratio of participants to independent variables was not sufficient.

### Paternal psychosocial work conditions

Exposure to job strain (i.e., work stress) can be measured in different ways. Typically work stress is assessed from self-reports via a questionnaire, inferred from occupational titles, or 'externally' assessed by expert job evaluators that evaluate the degree to which certain jobs are stressful on the basis of the job characteristics [42].

This study used both occupational titles and job evaluators to assess work stress. Specifically we obtained historical estimates of job control, psychological demand, physical demand, social support, and noise among the sawmill workers (i.e., the fathers) in the following way: 4 experienced job evaluators (two union and two management) in the British Columbia sawmill industry filled out the demand/control questionnaire to obtain a retrospective estimation for all basic job titles prior to 1975 (see Ostry, Marion, Green et al. [43], for these methods); 2) a panel of senior workers was selected in each participating mill and completed the demand/control questionnaire for basic job titles in their mill for two time periods (1975 to 1985), (1985 to 1998) (see Ostry, Marion, Demers et al. [44] for these methods). In the present study these expert estimates of fathers' psychosocial work conditions are treated as exposures to their offspring. Because estimates were provided for each sub-dimension of the demand/control model, we were able to investigate the association between specific aspects of fathers' work condition (i.e., control, psychological demand, physical demand, social support, and noise) and mental health outcomes in their offspring.

### Employment history, mental health, and socio-demographics

While the study focuses on the effect of paternal psychosocial working conditions on the development of psychopathology, there are some potential confounding variables that need to be accounted for in the analysis. Such variables are paternal socio-demographic characteristics, paternal mental health and paternal employment history.

From the job history records of the sawmill workers cohort we obtained the number of episodes of unemployment, job mobility (classified as upward, downward or stable), occupation (manager, tradesman, skilled worker, and unskilled worker) ethnicity, and marital status.

Mental health and alcohol dependence of the fathers were obtained from a provincial administrative database. Using probabilistic linkage techniques the sawmill workers' cohort was linked to the British Columbia Linked Health Database (BCLHDB), consisting of person-specific, longitudinal records on all British Columbians. The BCLHDB contains files with data on deaths, hospital discharges, and all physician encounters for the years 1985 through to 2001. The records are stored separately but have been indexed with an individual service-recipient specific code so that individual's records can be linked across files for specific research projects.

Ethical approval was obtained from the University of British Columbia (UBC) and the British Columbia Ministry of Health to conduct this study. A Data Access Subcommittee consisting of health ministry personnel, staff from the British Columbia Ministry of Information and Privacy, and the UBC Centre for Health Services and Policy Research has been established to handle requests for linkage to the BCLHDB and to ensure that such requests meet scientific and ethical standards, are in the public interest, and conform with the Freedom on Information and Protection of Privacy Act.

Number of episodes of unemployment, job mobility, occupation, ethnicity, marital status, alcohol dependence and mental health of the father were used in the analysis to control for the potential confounding effect of these variables on the association between psychosocial work conditions and developmental psychopathology.

### Mental Health Outcomes of the Children's Cohort

In British Columbia children who experience mental health problems can be evaluated by mental health professional at hospitals or medical clinics. The reason for medical visit or hospitalization (which can include a diagnosis if one is provided) is recorded on administrative forms that are sent and stored at the British Columbia Ministry of Health. In addition, complete hospital discharge and physician visit records for the paediatric and adult population are available through the BCLHDB that also provides the ICD9 codes for mental health conditions. The BCLHDB was accessed to identify all cases with a diagnosis of mental health occurring between Jan 1^st^, 1991 and March, 31st, 2001. We designated 1991 as the start year because it was the first year that ICD diagnoses were obtained on physician billing records in British Columbia. The same probabilistic linkage techniques outlined above were used here for the identification of mental health cases.

A mental health case was defined as an individual who had been assigned for the first time a mental health diagnosis *after *the start of the father's employment at one of the study sawmills. Furthermore, only mental health conditions that allowed comparisons to be made between at least three of the four age groups are reported in this study.

A mental health case for children *14 years old and younger *was defined as a child's first diagnosis labelled with ICD9 codes 300 (neurotic disorder), 301 (personality disorders), 308 (acute reaction to stress), 309 (adjustment reaction), and 311 (depressive disorder).

A mental health case for individuals *15 years old and older *was defined as an individual's first diagnosis labelled with ICD9 codes 300 (neurotic disorder), 301 (personality disorder), 302 (sexual deviations), 303 (alcohol dependence), 304 (drug dependence), 305 (non-dependent drug abuse), 308 (acute reaction to stress), 309 (adjustment reaction), and 311 (depressive disorder).

Each participant was assigned a code of 1 (i.e., had been diagnosed) or 0 (i.e., had not been diagnosed) for each of the above ICD9 codes. For discussion purposes, these mental health diagnoses were grouped into two categories: non-psychotic disorders and drug and alcohol related disorders. Non-psychotic disorders include those conditions that do not have a psychotic symptomatology and are primarily characterized by anxiety and depressive symptoms. These include neurotic disorder, personality disorder, acute reaction to stress, adjustment reaction and depression. Drug and alcohol related disorders include those conditions associate with drug and alcohol use and abuse such as alcohol dependence, drug dependence, and non-dependent drug abuse.

**Figure 1:**
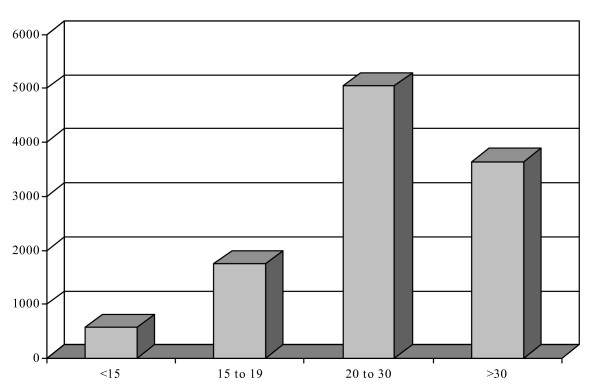
**Number of cases per age at diagnosis**. Figure 1 reveals that 67% of cases were identified by the age of 30, with young adulthood containing the greatest number of mental health diagnoses. The most prevalent diagnoses assigned to all age groups are neurotic disorders (children/early adolescents – 21%; adolescents – 20%; young adults – 20%; adults – 21%) and depression (children/early adolescents – 33%; adolescents – 37%; young adults – 33%; adults – 31%). The third most prevalent diagnosis among children/early adolescents (20%) and adolescents (11%) was adjustment reaction whereas the third most prevalent diagnosis among young adults (13%) and adults (13%) was acute reaction to stress.

Note that because we identified age at *first *diagnosis as our criterion for inclusion, the age groups are 'exclusive' as far as *specific *diagnoses are concerned. For example, a participant can only be assigned a diagnosis of depression for the first time once. Thus each participant will only be represented in the age group reflecting his or her age at time of diagnosis for that specific diagnosis. On the other hand, the same participants can be diagnosed with *different *mental health conditions at different times in their life or within the same age period. Because of the high co-morbidity in mental health, it is possible that the same participants appear twice or more in the analysis within any specific age group or across different age groups. Figure 1 shows the age distribution of first mental health diagnoses among the four groups of participants.

### Analysis

Using survival-time to case-control on STATA 8.0, three controls were selected for each mental health case matched on age and gender. Controls were chosen randomly with replacement from the set at risk. The set at risk were all the offspring of the sawmill worker's cohort, born between 1952 and 2001, whose father had worked in a study sawmill for at least one year during the first 16 years of the child's life. These could be anyone at risk who also satisfied the matching criteria who had not been diagnosed with a mental health condition at the time of diagnosis of the case.

Statistical analyses were conducted using conditional logistic regression on STATA 8.0. A total of 28 multivariate models were run (4 models for the children's cohort; 8 models for the adolescent cohort; 8 models for the young adult cohort; and 8 models for the adult cohort). The independent variables were the same for each of the 28 models tested and included: control (ordinal – lowest value as the referent); psychological demand (ordinal – lowest value as the referent); physical demand (ordinal – lowest value as the referent); social support (ordinal – lowest value as the referent); and noise (ordinal – lowest value as the referent).

Control variables included in the models were: duration of employment (continuous variable); paternal ethnicity (one dummy variable for Chinese and one dummy variable for Sikh – Caucasian was as referent); marital status (one dummy variable – married as the referent); paternal alcohol dependence (one dummy variable – no diagnosis as the referent); mental health of the father (one dummy variable – no diagnosis as the referent); suicidal behaviour of the father (one dummy variable – no diagnosis as the referent); type of employment (one dummy variable for trades, one dummy variable for skilled, and one dummy variable for unskilled – management was as referent); All independent variables and control variables were entered at once and no stepwise procedure was used.

## Results

Figure 1 indicates that 67% of cases were identified by the age of 30, with young adulthood representing the developmental period with the greatest number of first time mental health diagnoses. The most prevalent diagnoses assigned to all age groups are depression (children/early adolescents – 33%; adolescents – 37%; young adults – 33%; adults – 31%), and neurotic disorders (children/early adolescents – 21%; adolescents – 20%; young adults – 20%; adults – 21%). Adjustment reaction was the third most prevalent diagnosis among children/early adolescents (20%) and adolescents (11%); and acute reaction to stress was the third most prevalent diagnosis among young adults (13%) and adults (13%).

Table 2 and Additional file 1, 2 and 3 show the results of the multivariate analyses for children/early adolescents, adolescents, young adults, and adults respectively.

**Table 2:** Results of the multivariate analysis among the children and early adolescents' cohort

Predictor	Odds Ratio	SE	z	P > |z|	95% CI
*Neurotic Disorders*					
Duration of Employment	0.995	0.047	-0.11	0.912	0.907–1.09
Control	0.948	0.067	-0.75	0.451	0.826–1.09
Psychological Demand	1.05	0.109	0.46	0.644	0.856–1.29
Physical Demand	1.62	0.606	1.29	0.196	0.779–3.37
Social Support	1.06	0.278	0.20	0.839	0.629–1.77
Noise	0.899	0.363	-0.26	0.794	0.408–1.99
Trades Worker	4.31	4.80	1.31	0.189	0.486–38.2
Skilled Worker	3.79	4.19	1.21	0.228	0.435–33.1
Unskilled Worker	4.03	4.43	1.27	0.204	0.468–34.8
Marital Status	1.05	0.081	0.71	0.479	0.908–1.23
Chinese	0.459	0.517	-0.69	0.489	0.051–4.17
Sikh	0.193	0.093	-3.42	0.001*	0.075-.495
Paternal Alcoholism	0.308	0.365	-0.99	0.320	0.030–3.13
Paternal Mental Health	3.08	0.920	3.77	0.000*	1.72–5.53
Paternal Suicidal Behaviours	0.590	0.597	-0.52	0.602	0.081–4.28
*Acute Reaction to Stress*					
Duration of Employment	1.04	0.079	0.46	0.646	0.892–1.20
Control	0.955	0.122	-0.36	0.721	0.744–1.23
Psychological Demand	0.963	0.165	-0.22	0.827	0.688–1.35
Physical Demand	0.698	0.420	-0.60	0.550	0.214–2.27
Social Support	0.620	0.269	-1.10	0.271	0.265–1.45
Noise	1.26	0.865	0.34	0.735	0.329–4.84
Trades Worker	0.747	0.609	-0.36	0.721	0.151–3.69
Skilled Worker	0.436	0.368	-0.98	0.325	0.083–2.28
Unskilled Worker	1.20	0.964	0.23	0.818	0.250–5.79
Marital Status	1.06	0.109	0.59	0.555	0.869–1.30
Chinese	3.93e-16	2.33e-8	-0.00	1.00	0
Sikh	0.188	0.154	-2.04	0.041*	0.038-.935
Paternal Alcoholism	0.504	0.333	-1.04	0.299	0.138–1.84
Paternal Mental Health	1.60	0.679	1.10	0.271	0.694–3.68
Paternal Suicidal Behaviours	1.53e-15	6.32e-8	-0.00	1.00	0
*Adjustment Reaction*					
Duration of Employment	0.960	0.048	-0.80	0.421	0.869–1.06
Control	0.955	0.066	-0.65	0.515	0.834–1.09
Psychological Demand	0.838	0.100	-1.47	0.140	0.663–1.05
Physical Demand	1.28	0.520	0.62	0.536	0.580–2.84
Social Support	0.820	0.215	-0.75	0.452	0.489–1.37
Noise	0.706	0.262	-0.94	0.349	0.340–1.46
Trades Worker	0.755	0.614	-0.35	0.730	0.153–3.71
Skilled Worker	0.821	0.694	-0.23	0.816	0.156–4.30
Unskilled Worker	0.909	0.743	-0.12	0.908	0.183–4.51
Marital Status	1.00	0.072	0.08	0.934	0.873–1.15
Chinese	0.514	0.570	-0.60	0.549	0.058–4.52
Sikh	0.154	0.077	-3.71	0.000*	0.057-.414
Paternal Alcoholism	0.873	0.700	-0.17	0.866	0.181–4.20
Paternal Mental Health	2.04	0.568	2.57	0.010*	1.18–3.52
Paternal Suicidal Behaviours	0.874	1.19	-0.10	0.922	0.060–12.6
*Depression*					
Duration of Employment	0.991	0.037	-0.23	0.821	0.921–1.07
Control	0.934	0.050	-1.25	0.211	0.841–1.03
Psychological Demand	0.949	0.076	-0.64	0.522	0.811–1.11
Physical Demand	0.750	0.204	-1.05	0.293	0.440–1.28
Social Support	0.787	0.153	-1.22	0.222	0.537–1.15
Noise	0.685	0.200	-1.29	0.197	0.386–1.21
Trades Worker	0.672	0.359	-0.74	0.458	0.236–1.91
Skilled Worker	0.482	0.264	-1.33	0.184	0.164–1.41
Unskilled Worker	0.480	0.262	-1.34	0.179	0.165–1.39
Marital Status	1.00	0.077	0.07	0.943	0.865–1.16
Chinese	0.565	0.455	-0.71	0.479	0.116–2.74
Sikh	0.184	0.067	-4.60	0.000*	0.090-.379
Paternal Alcoholism	1.08	0.746	0.12	0.904	0.282–4.17
Paternal Mental Health	2.17	0.484	3.49	0.000*	1.40–3.36
Paternal Suicidal Behaviours	1.12	1.14	0.12	0.907	0.154–8.21

*p < .05

Ethnicity of the father was often associated with mental health conditions from childhood through adulthood. Children of fathers of Sikh or Chinese origin were consistently at lower risk of being diagnosed with mental health conditions such as depression, neurotic disorders, adjustment reaction, acute reaction to stress, and non-dependent drug abuse. Ethnic origin was associated with different age groups depending on the specific mental health condition. For example, being of Sikh origin functioned as a protective factor against depression and neurotic disorders among children (neurotic disorder: OR = .193, p = .001, 95% IC = .075–.495; depression: OR = .184, p < .001, 95%CI = .090–.379) and adolescents (neurotic disorder: OR = .288, p < .001, 95% IC = .185–.447; depression: OR = .439, p < .001, 95%CI = .331–.581), but not among young adults (neurotic disorder: OR = .830, p > .05, 95% IC = .663–1.04; depression: OR = .870, p > .05, 95%CI = .731–1.04) and adults (neurotic disorder: OR = .1.05, p > .05, 95% IC = .657–1.68; depression: OR = .953, p > .05, 95%CI = .652–1.39); and being of Chinese origin functioned as a protective factor for depression (OR = .211, p < .001, 95%CI = .114–.393) and neurotic disorders among young adults (OR = .357, p = .001, 95%CI = .193–.661), and for adjustment reaction among adolescents (OR = .187, p < .05, 95%CI = .038–.930) and young adults (OR = .186, p < .05, 95%CI = .043–.807).

Worth of noting is that while the proportion of Chinese participants in our sample (approximately 1% or less of the general population) is representative of the British Columbia population the Sikh participants are overrepresented in our sample with approximately 13% of the participants versus the expected 3% in the general BC population [45].

Duration of employment was associated with depression among adolescents (OR = .964, p < .05, 95%CI = .937–.991), alcohol dependence among adults (OR = .931, p < .05, 95%CI = .883–.982), and non-dependent drug abuse among adults (OR = .924, p < .05, 95%CI = .871–.979): the longer the fathers were employed at one sawmill the less the risk of being diagnosed with each of these conditions.

Paternal psychosocial work conditions were also significant factors associated with all mental health conditions investigated. However, different sub-dimensions of psychosocial work conditions were associated with specific diagnoses across the different age groups (see Table 2 and Additional files 1, 2, 3): control was associated with adjustment reaction among adolescents (OR = .866, p < .05, 95%CI = .775–.967) and alcohol dependence among adolescents (OR = .770, p < .05, 95%CI = .536–.994); psychological demand was associated with neurotic disorders among young adult (OR = .702, p < .001, 95%CI = .568–.868); noise was associated with acute reaction to stress among adults (OR = 1.42, p < .05, 95%CI = 1.06–1.91); and social support was associated with non-dependent drug abuse among adults (OR = 1.95, p < .05, 95%CI = 1.12–3.42).

## Discussion

In this study we investigated whether paternal psychosocial work conditions played a role in developmental psychopathology among the children of a cohort of sawmill workers from British Columbia, Canada. The present study has generated some important findings that contribute to the literature on mental health and developmental psychopathology in different ways.

First, we found that ethnicity of the father was a significant predictor where the risk of being diagnosed with one of several mental health outcomes from childhood to adulthood was lower for children of Chinese of Sikh fathers. This finding is consistent with what we found among adult sawmill workers where mental health diagnoses were less likely to be observed among Sikh and Chinese workers [46]. This finding is also consistent with findings from studies on the role of ethnicity on child development indicating that positive ethnic identity among minority children and adolescents functions as a protective factor for mental health and school outcomes [2-4].

Second, we found that the longer the fathers were employed at one sawmill the less the risk of being diagnosed with depression, alcohol dependence, and non-dependent drug abuse. This finding is consistent with the literature on unemployment and health in that is suggests that children may be affected by paternal unemployment and loss of income in important ways [24-28].

Third, we found that the association between paternal psychosocial work conditions (that in most cases were measured years prior to the mental health diagnosis) and mental health outcomes could be different depending on the age cohort (e.g., whether it was the children's or the adult's cohort). For example, with the exception of adjustment reaction, parental psychosocial work conditions played a more important role in adolescence and adulthood than it did during childhood. In some cases, paternal psychosocial work conditions were associated with mental health diagnoses in adulthood and not in other age groups such as in acute reaction to stress and non-dependent drug abuse. These findings suggest that while paternal psychosocial work conditions are significantly associated with a number of different mental health diagnoses, their effects are generally more noticeable over the long term than in the immediate future.

To a certain extent the results reported in this study are consistent with findings of the developmental literature in that they provide further evidence of the significant impact of early influences on health and development from childhood throughout adulthood. However, these findings have some important elements of novelty, most notably the original evidence of the critical role that exposure to adverse paternal psychosocial work conditions play in the first sixteen years of life contributing to the onset of mental health outcomes in different age cohorts.

While this study used rigorous design and analytical strategies, there are some limitations that need to be mentioned. One limitation is related to the use of an administrative database instead of complete clinical records to infer diagnosis which can lead to misclassification bias. However, this potential bias may be minimized because we used a large number of participants. Another important limitation of the study is the possible bias linked to the expected co-morbidity in mental health and the fact that the same participants may have appeared twice or more in the analysis.

At the conceptual level, a greater issue that constitutes a potentially important limitation is linked to the historical challenge of defining mental health and the ways in which mental health manifests in minority cultures. In the present study mental health was implicitly defined according to the parameters of psychiatry and clinical psychology traditions embodied in the DSM IV (and reflected in the ICD9 codes) where 'mental disease' rather than 'mental health' is identified. This is a very important issue especially in light of fact that important associations between ethnic origin and several mental health diagnoses were here identified. Vega and Rumbaut [47] eloquently describe the important issues that influence the way minority mental health is investigated in North American societies. Specifically, they argue that one of the greatest challenges of contemporary researchers lies in their ability to disentangle cultural influences from more accurate measurement and understanding of psychiatric problems in minority cultures.

Despite the potential limitations of this study, the findings reported here are overall important in many respects. First, they contribute to the advancement of theories of developmental psychopathology by incorporating concepts from the occupational health literature (i.e., psychosocial work conditions) and generating discussion on ways in which these can be 'brought home' and turned into 'adverse childhood experiences'. Second, results of this study also add to the literature of the effect of parental unemployment on child development and specifically identify what sub-dimensions of psychosocial work conditions are the most probable to increase the risk for developmental psychopathology from childhood to adulthood. Third, findings from this study may also have important clinical implications by informing age specific intervention strategies and family therapy approaches where parents are offered support to cope with work stress and related occupational challenges.

## Conclusion

By investigating several mental health outcomes of 19,833 children of sawmill workers from British Columbia, Canada we found that the degree to which children are exposed to adverse paternal psychosocial work conditions during childhood is associated with their mental health outcomes throughout adulthood. his study provides support to the tenet that being exposed to paternal work stress during childhood can have long lasting effects on the mental health of individuals.

## Competing interests

The author(s) declare that they have no competing interests.

## Authors' contributions

SM designed the analytical plan and conceived the conceptual framework of the manuscript; AO participated in the development of the analysis and conceptual framework. JT and JD reviewed drafts of the manuscript. RH and LC carried out the analysis. CH established the sawmill cohort, obtained linkages with health administrative data, and took part in drafting the manuscript. All authors read and approved the final manuscript.

## Pre-publication history

The pre-publication history for this paper can be accessed here:



## Supplementary Material

Additional file 1 Results of the multivariate analysis among the adolescent cohort. The data provided represent the multivariate analysis for the adolescent cohort. Click here for file

Additional file 2 Results of the multivariate analysis among the young adult cohort. The data provided represent the multivariate analysis for the young adult cohort. Click here for file

Additional file 3 Results of the multivariate analysis among the adult cohort. The data provided represent the multivariate analysis for the adult cohort. Click here for file

## References

[B1] Hughes D, Rodriguez J, Smith EP, Johnson DJ, Stevenson HC, Spicer P (2006). Parents' ethnic-racial socialization practices: a review of research and directions for future study. Developmental Psychology.

[B2] Chatman CM, Eccles JS, Malanchuk O, Downey G, Eccles JS, Chatman CM (2005). Identity negotiations in everyday settings. Navigating the future: social identity, coping and life tasks.

[B3] Oyserman D, Harrison K, Bybee D (2001). Can racial identity be promotive of academic efficacy?. International Journal of Behavioral Development.

[B4] Shelton JN, Yip T, Eccles JS, Chatman CM, Fuligni A, Wong C, Downey G, Eccles JS, Chatman CM (2005). Ethnic identity as a buffer of psychological adjustment to stress. Navigating the future: social identity, coping, and life tasks.

[B5] Cicchetti D, Toth SL, Cicchetti D, Toth SL (1991). A developmental perspective on internalizing and externalizing disorders. Internalizing and Externalizing Expression of Dysfunction.

[B6] Cicchetti D, Toth SL, Eds (1997). Developmental perspectives on trauma: theory, research and intervention.

[B7] Haggerty RJ, Sherrod LR, Garmezy N, Rutter M, Eds (1994). Stress, risk, and resilience in children and adolescents: processes, mechanisms, and interventions.

[B8] Rutter M (1989). Pathways from childhood to adult life. Journal of Child Psychology and Psychiatry.

[B9] Attar B, Guerra N, Tolan P (1994). Neighborhood disadvantage, stressful life events, and adjustment in urban elementary-school children. Journal of Clinical Child Psychology.

[B10] Kliewer W, Wolchik S, Sandler IN (1997). Children's coping with chronic illness. Handbook of children's coping: linking theory and intervention Issues in clinical child psychology.

[B11] Worsham N, Compas B, Ey S, Wolchik S, Sandler IN (1997). Children's coping with parental illness. Handbook of children's coping: linking theory and intervention Issues in clinical child psychology.

[B12] McLoyd VC, Wilson L, Huston AC (1991). The strain of living poor: parenting, social support, and child mental health. Children in poverty: child development and public policy.

[B13] Garmezy N, Garmezy N, Rutter M (1983). Stressors of childhood. Stress, coping, & development in children.

[B14] Conger RD, Conger K, Elder G, Lorenz F, Simons R, Whitbeck L (1992). A family process model of economic hardship and adjustment of early adolescent boys. Child Development.

[B15] Elder G, Nguyen T, Caspi A (1985). Linking family hardship to children's lives. Child Development.

[B16] Lempers J, Clark-Lempers D, Simons R (1989). Economic hardship, parenting, and distress in adolescence. Child Development.

[B17] Elder G (1974). Children of the Great Depression.

[B18] Eichorn DH, Clausen JA, Haan N, Honzik MMP (1981). PHM: Present and past in middle life.

[B19] Elder G, Liker J, Cross C, Baltes P, Brim Jr O (1984). Parent-child behavior in the Great-Depression: life course and intergenerational influences. Life Span development and behavior.

[B20] Liker JK, Elder GHJ (1983). Economic hardship and marital relations in the 1930s. American Sociological Review.

[B21] Edwards VJ, Holden GW, Felitti VJ, Anda RF (2003). Relationship between multiple forms of childhood maltreatment and adult mental health in community respondents: results from the adverse childhood experiences study. American Journal of Psychiatry.

[B22] Anda RF, Croft JB, Felitti VJ, Nordenberg D, Giles WH, Williamson DF, Giovino GA (1999). Adverse childhood experiences and smoking during adolescence and adulthood. JAMA.

[B23] Felitti VJ, Anda RF, Nordenberg D, Williamson DF, Spitz AM, Edwards V, Koss MP, Marks JS (1998). Relationship of childhood abuse and household dysfunction to many of the leading causes of death in adults: the adverse childhood experiences (ACE) Study. American Journal of Preventive Medicine.

[B24] Flanagan CA, Eccles JS (1993). Changes in parents' work status and adolescents' adjustment at school. Child Development.

[B25] Stewart W, Barling J (1996). Fathers' work experiences effect children's behaviors via job-related affect and parenting behaviors. Journal of Organizational Behavior.

[B26] Barling J, Zacharatos A, Hepburn CG (1999). Parents' job insecurity affects children's academic performance through cognitive difficulties. Journal of Applied Psychology.

[B27] Barling J, Dupre KE, Hepburn CG (1998). Effects of parents' job insecurity on children's work beliefs and attitudes. Journal of Applied Psychology.

[B28] MacEwan KE, Barling J (1991). Effects of maternal employment experiences on children's behavior via mood, cognitive difficulties, and parenting behavior. Journal of Marriage and Family.

[B29] Ostry A, Maggi S, Tansey J, Dunn J, Hershler R, Chen L, Louie AM, Hertzman C (2006). The impact of fathers' physical and psychosocial work conditions on attempted and completed suicide among their children. BMC Public Health.

[B30] Kessler RC, Berglund P, Demler O, Jin R, Walters EE (2005). Lifetime prevalence and age-of-onset distribution of DSM-IV disorders in the National Comorbidity Survey Replication. Archives of General Psychiatry.

[B31] Kilpatrick DG, Acierno R, Saunders B, Resnick HS, Best CL, Schnurr PP (2000). Risk factors for adolescent substance abuse and dependence: data from a national sample. Journal of Consulting and Clinical Psychology.

[B32] Kilpatrick DG, Ruggiero KJ, Acierno R, Saunders BE, Resnick HS, Best CL (2003). Violence and risk of PTSD, major depression, substance abuse/dependence, and co-morbidity: results from the National Survey of Adolescents. Journal of Consulting and Clinical Psychology.

[B33] Biederman J, Hirshfeld-Becker DR, Rosenbaum JF, Hérot C, Friedman D, Snidman N, Kagan J, Faraone SV (2001). Further evidence of association between behavioural inhibition and social anxiety in children. American Journal of Psychiatry.

[B34] U. S. Department of Health and Human Services, Administration on Children, Youth and Families (2001). In focus: Understanding the effects of maltreatment on early brain development.

[B35] Rutter M, Yule B, Quinton D, Rowlands O, Yule W, Berger M (1975). Attainment and adjustment in two geographical areas: III some factors accounting for area differences. British Journal of Psychiatry.

[B36] Karasek R, Theorell T (1990). Healthy work: stress, productivity, and the reconstruction of working life.

[B37] Karasek R, Theorell T, Schnall PL, Belkic K, Landsbergis P, Baker D (2000). The demand-control-support model and CVD. The workplace and cardiovascular disease.

[B38] Brooks-Gunn J, Auth JJ, Petersen AC, Compas BE, Goodyer IM (2001). Physiological processes and the development of childhood and adolescent depression. The depressed child and adolescent.

[B39] Gunnar MR (1998). Quality of early care and buffering of neuroendocrine stress reactions: potential effects on the developing brain. Preventive Medicine: an International Journal Devoted to Practice and Theory.

[B40] Leffert N, Petersen AC, Bornstein MH, Genevro JL (1996). Biology, challenge, and coping in adolescence: effects on physical and mental health. Child development and behavioral pediatrics: crosscurrents in contemporary psychology.

[B41] Hertzman C, Teschke K, Ostry A, Hershler R, Dimich-Ward H, Kelly S (1997). Mortality and cancer incidence among sawmill workers exposed to chlorophenate wood preservatives. American Journal of Public Health.

[B42] Belkic KL, Landsbergis PA, Schnall PL, Baker D (2004). Is job strain a major source of cardiovascular disease risk?. Scandinavian Journal of Work Environment and Health.

[B43] Ostry A, Marion SA, Green L, Demers PA, Hershler R, Kelly S (2001). Comparison of expert-rater methods for assessing psychosocial job strain. Scandinavian Journal of Work Environment and Health.

[B44] Ostry A, Marion SA, Demers PA, Hershler R, Kelly S, Teschke K (2001). Measuring psychosocial job strain with the job content questionnaire using experienced job evaluators. American Journal of Industrial Medicine.

[B45] Statistics Canada. n.d Population by selected ethnic origins, by province and territory (Table) British Columbia.

[B46] Ostry A, Maggi S, Tansey J, Dunn J, Hershler R, Chen L, Louie AM, Hertzman C The impact of psychosocial work conditions on attempted and completed suicide among Western Canadian sawmill workers. Scandinavian Journal of Public Health.

[B47] Vega WA, Rumbaut RG (1991). Ethnic minorities and mental health. Annual Review of Sociology.

