# Supplementary material for: Paternal psychosocial work conditions and mental health outcomes: A case-control study
Source: BMC Public Health. 2008 Mar 31;8:104. doi: 10.1186/1471-2458-8-104 (PMC2358891; doi:10.1186/1471-2458-8-104)
Supplement: Additional file 1 — Results of the multivariate analysis among the adolescent cohort. The data provided represent the multivariate analysis for the adolescent cohort. [file 1471-2458-8-104-S1.doc]

**Table 3 -** Results of the multivariate analysis among the adolescent cohort

| Predictor | Odds Ratio | SE | z | P> |z| | 95% CI |
| --- | --- | --- | --- | --- | --- |
| *Neurotic Disorders* |  |  |  |  |  |
| Duration of Employment | 1.00 | 0.020 | 0.08 | 0.936 | 0.964-1.04 |
| Control | 0.975 | 0.037 | -0.66 | 0.509 | 0.905-1.05 |
| Psychological Demand | 1.03 | 0.044 | 0.86 | 0.392 | 0.954-1.13 |
| Physical Demand | 0.956 | 0.212 | -0.20 | 0.838 | 0.618-1.48 |
| Social Support | 1.12 | 0.156 | 0.81 | 0.420 | 0.851-1.47 |
| Noise | 0.776 | 0.179 | -1.10 | 0.272 | 0.494-1.22 |
| Trades Worker | 1.41 | 0.538 | 0.89 | 0.371 | 0.665-2.98 |
| Skilled Worker | 2.14 | 0.831 | 1.97 | 0.049* | 1.00-4.58 |
| Unskilled Worker | 1.90 | 0.729 | 1.66 | 0.097 | 0.891-4.03 |
| Marital Status | 1.00 | 0.046 | 0.01 | 0.992 | 0.914-1.09 |
| Chinese | 0.610 | 0.309 | -0.98 | 0.329 | 0.226-1.65 |
| Sikh | 0.288 | 0.065 | -5.54 | 0.000* | 0.185-.447 |
| Paternal Alcoholism | 0.620 | 0.189 | -1.57 | 0.116 | 0.342-1.13 |
| Paternal Mental Health | 0.717 | 0.145 | -1.65 | 0.100 | 0.483-1.07 |
| Paternal Suicidal Behaviours | 4.14 | 2.33 | 2.53 | 0.011* | 1.38-12.5 |
| *Personality Disorders* |  |  |  |  |  |
| Duration of Employment | 0.993 | 0.046 | -0.13 | 0.895 | 0.908-1.09 |
| Control | 0.869 | 0.072 | -1.69 | 0.092 | 0.738-1.02 |
| Psychological Demand | 1.00 | 0.094 | 0.02 | 0.985 | 0.833-1.20 |
| Physical Demand | 2.05 | 0.939 | 1.56 | 0.118 | 0.833-5.03 |
| Social Support | 1.15 | 0.339 | 0.46 | 0.643 | 0.643-2.05 |
| Noise | 0.554 | 0.277 | -1.18 | 0.237 | 0.208-1.47 |
| Trades Worker | 0.682 | 0.568 | -0.46 | 0.646 | 0.133-3.49 |
| Skilled Worker | 1.86 | 1.58 | 0.73 | 0.466 | 0.352-9.81 |
| Unskilled Worker | 0.694 | 0.591 | -0.43 | 0.668 | 0.131-3.68 |
| Marital Status | 0.826 | 0.098 | -1.60 | 0.109 | 0.654-1.04 |
| Chinese | 0.333 | 0.318 | -1.15 | 0.249 | 0.051-2.16 |
| Sikh | 0.226 | 0.112 | -2.99 | 0.003* | 0.086-0.60 |
| Paternal Alcoholism | 0.493 | 0.301 | -1.16 | 0.247 | 0.149-1.63 |
| Paternal Mental Health | 0.904 | 0.389 | -0.23 | 0.815 | 0.389-2.10 |
| Paternal Suicidal Behaviours | 2.50e+15 | 4.59e+22 | 0.00 | 1.00 | 0 |
| *Acute Reaction to Stress* |  |  |  |  |  |
| Duration of Employment | 0.976 | 0.026 | -0.89 | 0.371 | 0.926-1.03 |
| Control | 0.951 | 0.047 | -1.02 | 0.309 | 0.862-1.05 |
| Psychological Demand | 0.991 | 0.057 | -0.15 | 0.882 | 0.885-1.11 |
| Physical Demand | 0.914 | 0.279 | -0.29 | 0.770 | 0.504-1.66 |
| Social Support | 1.22 | 0.228 | 1.06 | 0.290 | 0.845-1.76 |
| Noise | 1.60 | 0.523 | 1.44 | 0.151 | 0.843-3.04 |
| Trades Worker | 1.06 | 0.553 | 0.12 | 0.904 | 0.385-2.94 |
| Skilled Worker | 1.58 | 0.931 | 0.87 | 0.386 | 0.563-4.43 |
| Unskilled Worker | 1.23 | 0.659 | 0.38 | 0.702 | 0.429-3.51 |
| Marital Status | 0.999 | 0.053 | -0.01 | 0.992 | 0.902-1.11 |
| Chinese | 1.14 | 0.799 | 0.18 | 0.854 | 0.287-4.51 |
| Sikh | 0.360 | 0.104 | -3.53 | 0.000* | 0.204-0.64 |
| Paternal Alcoholism | 0.748 | 0.453 | -0.48 | 0.632 | 0.229-2.45 |
| Paternal Mental Health | 1.43 | 0.309 | 1.67 | 0.096 | 0.939-2.19 |
| Paternal Suicidal Behaviours | 2.41 | 1.72 | 1.24 | 0.215 | 0.599-9.74 |
| *Adjustment Reaction* |  |  |  |  |  |
| Duration of Employment | 0.986 | 0.029 | -0.49 | 0.627 | 0.931-1.04 |
| Control | 0.866 | 0.049 | -2.55 | 0.011* | 0.775-.967 |
| Psychological Demand | 0.985 | 0.064 | -0.23 | 0.818 | 0.867-1.12 |
| Physical Demand | 0.989 | 0.297 | -0.04 | 0.970 | 0.549-1.78 |
| Social Support | 1.09 | 0.201 | 0.45 | 0.656 | 0.755-1.56 |
| Noise | 1.66 | 0.507 | 1.65 | 0.098 | 0.910-3.02 |
| Trades Worker | 1.68 | 0.821 | 1.06 | 0.287 | 0.645-4.38 |
| Skilled Worker | 2.40 | 1.14 | 1.84 | 0.066 | 0.943-6.10 |
| Unskilled Worker | 1.54 | 0.722 | 0.91 | 0.361 | 0.612-3.86 |
| Marital Status | 1.05 | 0.053 | 1.09 | 0.276 | 0.957-1.17 |
| Chinese | 0.187 | 0.153 | -2.05 | 0.041* | 0.038-0.93 |
| Sikh | 0.284 | 0.086 | -4.15 | 0.000* | 0.157-0.52 |
| Paternal Alcoholism | 0.325 | 0.138 | -2.66 | 0.008* | 0.142-0.75 |
| Paternal Mental Health | 0.936 | 0.232 | -0.27 | 0.790 | 0.575-1.52 |
| Paternal Suicidal Behaviours | 2.48 | 1.62 | 1.39 | 0.164 | 0.691-8.94 |
| *Depression* |  |  |  |  |  |
| Duration of Employment | 0.964 | 0.014 | -2.59 | 0.010* | 0.937-0.99 |
| Control | 0.965 | 0.026 | -1.30 | 0.193 | 0.915-1.02 |
| Psychological Demand | 1.04 | 0.032 | 1.43 | 0.152 | 0.984-1.11 |
| Physical Demand | 1.30 | 0.200 | 1.67 | 0.094 | 0.957-1.75 |
| Social Support | 1.01 | 0.095 | 0.13 | 0.898 | 0.842-1.22 |
| Noise | 0.945 | 0.150 | -.035 | 0.723 | 0.693-1.29 |
| Trades Worker | 1.17 | 0.306 | 0.59 | 0.552 | 0.699-1.95 |
| Skilled Worker | 1.40 | 0.373 | 1.27 | 0.205 | 0.832-2.36 |
| Unskilled Worker | 0.970 | 0.266 | -0.11 | 0.912 | 0.568-1.66 |
| Marital Status | 1.00 | 0.029 | 0.15 | 0.884 | 0.949-1.06 |
| Chinese | 0.501 | 0.202 | -1.71 | 0.086 | 0.227-1.10 |
| Sikh | 0.439 | 0.063 | -5.75 | 0.000* | 0.331-0.58 |
| Paternal Alcoholism | 1.77 | 0.513 | 1.97 | 0.049* | 1.00-3.12 |
| Paternal Mental Health | 1.42 | 0.167 | 2.97 | 0.003* | 1.13-1.79 |
| Paternal Suicidal Behaviours | 1.40 | 0.482 | 0.97 | 0.330 | 0.712-2.75 |
| *Alcohol Dependence* |  |  |  |  |  |
| Duration of Employment | 0.865 | 0.073 | -1.72 | 0.085 | 0.733-1.02 |
| Control | 0.730 | 0.115 | -2.00 | 0.046* | 0.536-0.99 |
| Psychological Demand | 1.15 | 0.173 | 0.96 | 0.338 | 0.861-1.54 |
| Physical Demand | 0.559 | 0.433 | -0.75 | 0.453 | 0.122-2.55 |
| Social Support | 0.891 | 0.441 | -0.23 | 0.816 | 0.338-2.35 |
| Noise | 0.828 | 0.684 | -0.23 | 0.819 | 0.164-4.18 |
| Trades Worker | 1.43 | 2.64 | 0.19 | 0.846 | 0.038-53.2 |
| Skilled Worker | 1.39 | 2.48 | 0.18 | 0.854 | 0.042-46.2 |
| Unskilled Worker | 1.06 | 1.90 | 0.03 | 0.973 | 0.032-35.1 |
| Marital Status | 1.05 | 0.125 | 0.41 | 0.684 | 0.831-1.33 |
| Chinese | 3.32e-15 | 7.21e-8 | -0.00 | 1.00 | 0 |
| Sikh | 0.093 | 0.107 | -2.06 | 0.039* | 0.010-0.89 |
| Paternal Alcoholism | 0.770 | 1.34 | -0.15 | 0.881 | 0.025-23.5 |
| Paternal Mental Health | 0.245 | 0.287 | -1.20 | 0.229 | 0.025-2.42 |
| Paternal Suicidal Behaviours | 4.30 | 7.16 | 0.88 | 0.381 | 0.16-112.6 |
| *Drug Dependence* |  |  |  |  |  |
| Duration of Employment | 1.04 | 0.057 | 0.75 | 0.456 | 0.935-1.16 |
| Control | 0.793 | 0.099 | -1.86 | 0.062 | 0.621-1.01 |
| Psychological Demand | 1.09 | 0.141 | 0.69 | 0.490 | 0.849-1.41 |
| Physical Demand | 0.275 | 0.167 | -2.12 | 0.034* | 0.084-.907 |
| Social Support | 1.20 | 0.523 | 0.43 | 0.668 | 0.514-2.82 |
| Noise | 0.955 | 0.532 | -0.08 | 0.934 | 0.320-2.85 |
| Trades Worker | 2.21 | 2.29 | 0.77 | 0.442 | 0.293-16.7 |
| Skilled Worker | 0.644 | 0.631 | -0.45 | 0.653 | 0.095-4.39 |
| Unskilled Worker | 1.30 | 1.28 | 0.27 | 0.787 | 0.191-8.90 |
| Marital Status | 0.909 | 0.119 | -0.73 | 0.467 | 0.704-1.17 |
| Chinese | 0.707 | 0.881 | -0.28 | 0.781 | 0.061-8.14 |
| Sikh | 0.197 | 0.122 | -2.62 | 0.009* | 0.058-0.67 |
| Paternal Alcoholism | 0.310 | 0.272 | -1.34 | 0.182 | 0.056-1.73 |
| Paternal Mental Health | 0.601 | 0.304 | -1.01 | 0.314 | 0.222-1.62 |
| Paternal Suicidal Behaviours | 3.28e-15 | 7.13e-8 | 0.00 | 1.00 | 0 |
| *Non-Dependent Drug Abuse* |  |  |  |  |  |
| Duration of Employment | 1.07 | 0.053 | 1.43 | 0.153 | 0.974-1.18 |
| Control | 0.888 | 0.080 | -1.33 | 0.184 | 0.744-1.06 |
| Psychological Demand | 1.04 | 0.109 | 0.41 | 0.680 | 0.851-1.28 |
| Physical Demand | 1.43 | 0.750 | 0.69 | 0.493 | 0.513-4.00 |
| Social Support | 0.492 | 0.169 | -2.06 | 0.039* | 0.251-0.97 |
| Noise | 1.61 | 0.868 | 0.88 | 0.380 | 0.557-4.63 |
| Trades Worker | 0.899 | 0.776 | -0.12 | 0.902 | 0.166-4.88 |
| Skilled Worker | 0.431 | 0.351 | -1.03 | 0.301 | 0.087-2.13 |
| Unskilled Worker | 0.936 | 0.806 | -0.08 | 0.939 | 0.173-5.06 |
| Marital Status | 1.13 | 0.091 | 1.53 | 0.126 | 0.966-1.33 |
| Chinese | 1.18e-16 | 2.86e-9 | -0.00 | 1.00 | 0 |
| Sikh | 0.135 | 0.078 | -3.46 | 0.001* | 0.043-0.42 |
| Paternal Alcoholism | 1.64 | 1.85 | 0.44 | 0.660 | 0.180-15.0 |
| Paternal Mental Health | 1.89 | 0.730 | 1.65 | 0.100 | 0.886-4.03 |
| Paternal Suicidal Behaviours | 2.54 | 2.60 | 0.91 | 0.360 | 0.344-18.8 |

*p < .05
