# Supplementary material for: Paternal psychosocial work conditions and mental health outcomes: A case-control study
Source: BMC Public Health. 2008 Mar 31;8:104. doi: 10.1186/1471-2458-8-104 (PMC2358891; doi:10.1186/1471-2458-8-104)
Supplement: Additional file 2 — Results of the multivariate analysis among the young adult cohort. The data provided represent the multivariate analysis for the young adult cohort. [file 1471-2458-8-104-S2.doc]

**Table 4 -** Results of the multivariate analysis among the young adult cohort

| Predictor | Odds Ratio | SE | z | P> |z| | 95% CI |
| --- | --- | --- | --- | --- | --- |
| *Neurotic Disorders* |  |  |  |  |  |
| Duration of Employment | 0.989 | 0.010 | -1.09 | 0.278 | 0.969-1.01 |
| Control | 0.993 | 0.021 | -0.35 | 0.727 | 0.952-1.04 |
| Psychological Demand | 0.981 | 0.026 | -0.74 | 0.461 | 0.931-1.03 |
| Physical Demand | 1.24 | 0.153 | 1.77 | 0.077 | 0.976-1.58 |
| Social Support | 0.980 | 0.076 | -0.26 | 0.795 | 0.842-1.14 |
| Noise | 0.958 | 0.108 | -0.39 | 0.700 | 0.768-1.19 |
| Trades Worker | 0.881 | 0.155 | -0.72 | 0.472 | 0.624-1.24 |
| Skilled Worker | 1.13 | 0.213 | 0.63 | 0.529 | 0.777-1.63 |
| Unskilled Worker | 1.03 | 0.197 | 0.15 | 0.879 | 0.708-1.50 |
| Marital Status | 1.02 | 0.019 | 1.24 | 0.217 | 0.987-1.06 |
| Chinese | 0.357 | 0.112 | -3.28 | 0.001* | 0.193-0.66 |
| Sikh | 0.830 | 0.095 | -1.62 | 0.106 | 0.663-1.04 |
| Paternal Alcoholism | 0.767 | 0.131 | -1.55 | 0.121 | 0.548-1.07 |
| Paternal Mental Health | 0.661 | 0.075 | -3.65 | 0.000* | 0.529-0.83 |
| Paternal Suicidal Behaviours | 1.43 | 0.610 | 0.83 | 0.407 | 0.616-3.30 |
| *Personality Disorders* |  |  |  |  |  |
| Duration of Employment | 0.969 | 0.026 | -1.19 | 0.235 | 0.919-1.02 |
| Control | 1.03 | 0.056 | 0.47 | 0.640 | 0.922-1.14 |
| Psychological Demand | 0.941 | 0.062 | -0.92 | 0.357 | 0.827-1.07 |
| Physical Demand | 1.62 | 0.521 | 1.51 | 0.132 | 0.864-3.04 |
| Social Support | 0.799 | 0.168 | -1.07 | 0.286 | 0.529-1.21 |
| Noise | 0.901 | 0.282 | -0.33 | 0.739 | 0.487-1.67 |
| Trades Worker | 0.643 | 0.292 | -0.97 | 0.331 | 0.265-1.56 |
| Skilled Worker | 0.639 | 0.311 | -0.92 | 0.358 | 0.246-1.66 |
| Unskilled Worker | 0.795 | 0.376 | -0.48 | 0.628 | 0.315-2.01 |
| Marital Status | 0.997 | 0.048 | -0.07 | 0.944 | 0.906-1.10 |
| Chinese | 0.192 | 0.205 | -1.55 | 0.122 | 0.024-1.56 |
| Sikh | 0.647 | 0.196 | -1.44 | 0.150 | 0.357-1.17 |
| Paternal Alcoholism | 0.278 | 0.133 | -2.68 | 0.007* | 0.109-0.71 |
| Paternal Mental Health | 1.01 | 0.288 | 0.03 | 0.980 | 0.576-1.76 |
| Paternal Suicidal Behaviours | 5.53e-16 | 1.72e-8 | 0.00 | 1.00 | 0 |
| *Acute Reaction to Stress* |  |  |  |  |  |
| Duration of Employment | 0.991 | 0.013 | -0.71 | 0.479 | 0.966-1.02 |
| Control | 0.988 | 0.027 | -0.45 | 0.651 | 0.935-1.04 |
| Psychological Demand | 0.993 | 0.033 | -0.21 | 0.836 | 0.930-1.06 |
| Physical Demand | 1.38 | 0.219 | 2.04 | 0.042* | 1.01-1.88 |
| Social Support | 1.08 | 0.111 | 0.78 | 0.433 | 0.887-1.32 |
| Noise | 0.880 | 0.123 | -0.92 | 0.359 | 0.669-1.16 |
| Trades Worker | 0.844 | 0.191 | -0.75 | 0.454 | 0.542-1.31 |
| Skilled Worker | 1.36 | 0.329 | 1.29 | 0.198 | 0.850-2.19 |
| Unskilled Worker | 1.15 | 0.280 | 0.58 | 0.564 | 0.715-1.85 |
| Marital Status | 0.991 | 0.025 | -0.36 | 0.723 | 0.942-1.04 |
| Chinese | 0.135 | 0.080 | -3.36 | 0.001* | 0.042-0.43 |
| Sikh | 0.616 | 0.096 | -3.10 | 0.002* | 0.454-0.84 |
| Paternal Alcoholism | 1.48 | 0.402 | 1.43 | 0.154 | 0.864-2.52 |
| Paternal Mental Health | 1.49 | 0.174 | 3.45 | 0.001* | 1.19-1.88 |
| Paternal Suicidal Behaviours | 0.913 | 0.388 | -0.21 | 0.830 | 0.397-2.10 |
| *Adjustment Reaction* |  |  |  |  |  |
| Duration of Employment | 1.00 | 0.016 | 0.11 | 0.910 | 0.970-1.03 |
| Control | 0.965 | 0.035 | -0.98 | 0.327 | 0.899-1.04 |
| Psychological Demand | 0.993 | 0.043 | -0.15 | 0.881 | 0.912-1.08 |
| Physical Demand | 1.16 | 0.238 | 0.76 | 0.448 | 0.783-1.74 |
| Social Support | 1.22 | 0.156 | 1.54 | 0.124 | 0.947-1.57 |
| Noise | 0.873 | 0.155 | -0.76 | 0.444 | 0.618-1.24 |
| Trades Worker | 0.931 | 0.264 | -0.25 | 0.801 | 0.534-1.62 |
| Skilled Worker | 1.23 | 0.367 | 0.69 | 0.488 | 0.686-2.21 |
| Unskilled Worker | 1.01 | 0.306 | 0.04 | 0.964 | 0.561-1.83 |
| Marital Status | 1.03 | 0.035 | 1.02 | 0.306 | 0.969-1.10 |
| Chinese | 0.186 | 0.139 | -2.25 | 0.025* | 0.043-0.81 |
| Sikh | 0.663 | 0.131 | -2.08 | 0.038* | 0.450-0.98 |
| Paternal Alcoholism | 0.618 | 0.191 | -1.55 | 0.120 | 0.337-1.13 |
| Paternal Mental Health | 0.469 | 0.090 | -3.95 | 0.000* | 0.322-0.68 |
| Paternal Suicidal Behaviours | 2.50 | 1.79 | 1.28 | 0.199 | 0.617-10.1 |
| *Depression* |  |  |  |  |  |
| Duration of Employment | 0.971 | 0.008 | -3.67 | 0.000* | 0.955-0.99 |
| Control | 0.986 | 0.017 | -0.83 | 0.406 | 0.953-1.02 |
| Psychological Demand | 1.01 | 0.021 | 0.55 | 0.585 | 0.971-1.05 |
| Physical Demand | 1.03 | 0.099 | 0.26 | 0.794 | 0.849-1.24 |
| Social Support | 0.974 | 0.061 | -0.42 | 0.677 | 0.863-1.10 |
| Noise | 0.966 | 0.083 | -0.40 | 0.688 | 0.816-1.14 |
| Trades Worker | 0.914 | 0.123 | -0.67 | 0.505 | 0.703-1.19 |
| Skilled Worker | 1.13 | 0.166 | 0.84 | 0.401 | 0.848-1.51 |
| Unskilled Worker | 1.04 | 0.152 | 0.29 | 0.775 | 0.783-1.39 |
| Marital Status | 1.01 | 0.015 | 0.44 | 0.658 | 0.978-1.04 |
| Chinese | 0.211 | 0.067 | -4.92 | 0.000* | 0.114-0.39 |
| Sikh | 0.870 | 0.078 | -1.55 | 0.120 | 0.731-1.04 |
| Paternal Alcoholism | 1.58 | 0.291 | 2.46 | 0.014* | 1.10-2.26 |
| Paternal Mental Health | 1.28 | 0.097 | 3.26 | 0.001* | 1.10-1.48 |
| Paternal Suicidal Behaviours | 1.16 | 0.369 | 0.46 | 0.643 | 0.621-1.16 |
| *Alcohol Dependence* |  |  |  |  |  |
| Duration of Employment | 1.01 | 0.028 | 0.19 | 0.847 | 0.953-1.06 |
| Control | 1.02 | 0.064 | 0.25 | 0.803 | 0.898-1.15 |
| Psychological Demand | 0.903 | 0.067 | -1.37 | 0.170 | 0.780-1.04 |
| Physical Demand | 0.830 | 0.290 | -0.53 | 0.594 | 0.419-1.64 |
| Social Support | 1.10 | 0.234 | 0.46 | 0.646 | 0.727-1.67 |
| Noise | 1.42 | 0.428 | 1.16 | 0.244 | 0.787-2.56 |
| Trades Worker | 2.49 | 1.41 | 1.61 | 0.108 | 0.819-7.56 |
| Skilled Worker | 2.90 | 1.77 | 1.74 | 0.081 | 0.876-9.57 |
| Unskilled Worker | 3.32 | 2.01 | 1.98 | 0.047* | 1.01-10.8 |
| Marital Status | 0.969 | 0.051 | -0.60 | 0.548 | 0.873-1.07 |
| Chinese | 0.395 | 0.316 | -1.16 | 0.246 | 0.082-1.90 |
| Sikh | 0.705 | 0.229 | -1.08 | 0.282 | 0.373-1.33 |
| Paternal Alcoholism | 0.776 | 0.340 | -0.58 | 0.563 | 0.329-1.83 |
| Paternal Mental Health | 0.633 | 0.188 | -1.54 | 0.124 | 0.353-1.13 |
| Paternal Suicidal Behaviours | 0.883 | 1.01 | -0.11 | 0.913 | 0.094-8.29 |
| *Drug Dependence* |  |  |  |  |  |
| Duration of Employment | 0.991 | 0.021 | -0.42 | 0.678 | 0.951-1.03 |
| Control | 0.904 | 0.042 | -2.19 | 0.028* | 0.826-0.99 |
| Psychological Demand | 0.990 | 0.051 | -0.19 | 0.850 | 0.895-1.10 |
| Physical Demand | 0.648 | 0.170 | -1.66 | 0.098 | 0.387-1.08 |
| Social Support | 1.32 | 0.218 | 1.65 | 0.098 | 0.951-1.82 |
| Noise | 1.03 | 0.241 | 0.11 | 0.913 | 0.648-1.63 |
| Trades Worker | 1.13 | 0.382 | 0.37 | 0.710 | 0.586-2.19 |
| Skilled Worker | 1.19 | 0.435 | 0.46 | 0.642 | 0.578-2.43 |
| Unskilled Worker | 1.01 | 0.374 | 0.04 | 0.972 | 0.491-2.09 |
| Marital Status | 1.03 | 0.040 | 0.65 | 0.516 | 0.950-1.11 |
| Chinese | 0.304 | 0.232 | -1.56 | 0.119 | 0.068-1.36 |
| Sikh | 0.732 | 0.172 | -1.33 | 0.183 | 0.462-1.16 |
| Paternal Alcoholism | 0.421 | 0.150 | -2.42 | 0.015* | 0.209-0.85 |
| Paternal Mental Health | 0.944 | 0.216 | -0.25 | 0.802 | 0.603-1.48 |
| Paternal Suicidal Behaviours | 2.92 | 3.07 | 1.02 | 0.307 | 0.374-22.9 |
| *Non-Dependent Drug Abuse* |  |  |  |  |  |
| Duration of Employment | 0.977 | 0.024 | -0.96 | 0.340 | 0.930-1.03 |
| Control | 1.07 | 0.058 | 1.34 | 0.181 | 0.967-1.19 |
| Psychological Demand | 0.948 | 0.059 | -0.85 | 0.393 | 0.840-1.07 |
| Physical Demand | 1.44 | 0.425 | 1.24 | 0.214 | 0.810-2.57 |
| Social Support | 0.820 | 0.156 | -1.04 | 0.297 | 0.564-1.19 |
| Noise | 1.10 | 0.307 | 0.35 | 0.728 | 0.638-1.90 |
| Trades Worker | 0.908 | 0.419 | -0.21 | 0.835 | 0.368-2.24 |
| Skilled Worker | 1.20 | 0.602 | 0.36 | 0.723 | 0.446-3.21 |
| Unskilled Worker | 2.00 | 0.978 | 1.42 | 0.154 | 0.770-5.21 |
| Marital Status | 1.01 | 0.045 | 0.32 | 0.749 | 0.930-1.11 |
| Chinese | 0.342 | 0.278 | -1.32 | 0.187 | 0.069-1.69 |
| Sikh | 0.327 | 0.099 | -3.68 | 0.000* | 0.180-0.59 |
| Paternal Alcoholism | 1.38 | 0.681 | 0.65 | 0.519 | 0.522-3.63 |
| Paternal Mental Health | 1.45 | 0.322 | 1.65 | 0.098 | 0.934-2.24 |
| Paternal Suicidal Behaviours | 1.76 | 1.76 | 0.57 | 0.572 | 0.248-12.5 |

*p < .05
