# Supplementary material for: Paternal psychosocial work conditions and mental health outcomes: A case-control study
Source: BMC Public Health. 2008 Mar 31;8:104. doi: 10.1186/1471-2458-8-104 (PMC2358891; doi:10.1186/1471-2458-8-104)
Supplement: Additional file 3 — Results of the multivariate analysis among the adult cohort. The data provided represent the multivariate analysis for the adult cohort. [file 1471-2458-8-104-S3.doc]

**Table 5 -** Results of the multivariate analysis among the adult cohort

| Predictor | Odds Ratio | SE | z | P> |z| | 95% CI |
| --- | --- | --- | --- | --- | --- |
| *Neurotic Disorders* |  |  |  |  |  |
| Duration of Employment | .993 | .011 | -0.61 | .545 | .972-1.02 |
| Control | .972 | .026 | -1.07 | .283 | .924-1.02 |
| Psychological Demand | 1.05 | .081 | 0.60 | .549 | .900-1.22 |
| Physical Demand | 1.05 | .153 | 0.34 | .738 | .788-1.40 |
| Social Support | 1.08 | .103 | 0.82 | .413 | .897-1.30 |
| Noise | 1.01 | .117 | 0.07 | .941 | .804-1.27 |
| Trades Worker | 1.10 | .224 | 0.47 | .636 | .739-1.64 |
| Skilled Worker | 1.28 | .302 | 1.04 | .300 | .804-2.03 |
| Unskilled Worker | 1.07 | .256 | 0.29 | .772 | .671-1.71 |
| Marital Status | 1.00 | .025 | 0.20 | .843 | .957-1.05 |
| Chinese | .269 | .129 | -2.75 | .006* | .106-.687 |
| Sikh | 1.05 | .252 | 0.21 | .835 | .657-1.68 |
| Paternal Alcoholism | .615 | .140 | -2.13 | .033* | .393-.961 |
| Paternal Mental Health | .616 | .069 | -4.30 | .000* | .494-.768 |
| Paternal Suicidal Behaviours | .747 | .477 | -0.46 | .648 | .214-2.61 |
| *Personality Disorders* |  |  |  |  |  |
| Duration of Employment | .974 | .024 | -1.09 | .278 | .929-1.02 |
| Control | .982 | .054 | -0.34 | .737 | .882-1.09 |
| Psychological Demand | .870 | .161 | -0.75 | .451 | .605-1.25 |
| Physical Demand | 1.21 | .384 | 0.61 | .543 | .652-2.25 |
| Social Support | .930 | .188 | -0.36 | .721 | .626-1.38 |
| Noise | .769 | .194 | -1.04 | .298 | .469-1.26 |
| Trades Worker | 1.00 | .452 | 0.00 | .998 | .413-2.43 |
| Skilled Worker | 1.03 | .546 | 0.06 | .951 | .367-2.91 |
| Unskilled Worker | .767 | .393 | -0.52 | .604 | .281-2.09 |
| Marital Status | .972 | .056 | -0.50 | .619 | .869-1.09 |
| Chinese | 1.92e-15 | 2.09e-8 | -0.00 | 1.00 | 0 |
| Sikh | .496 | .290 | -1.20 | .230 | .158-1.56 |
| Paternal Alcoholism | .348 | .203 | -1.81 | .070 | .111-1.09 |
| Paternal Mental Health | .682 | .163 | -1.61 | .108 | .427-1.09 |
| Paternal Suicidal Behaviours | 1.90 | 1.78 | 0.69 | .491 | .304-11.91 |
| *Acute Reaction to Stress* |  |  |  |  |  |
| Duration of Employment | .995 | .014 | -0.38 | .704 | .968-1.02 |
| Control | 1.03 | .033 | 0.47 | .635 | .952-1.08 |
| Psychological Demand | .702 | .076 | -3.27 | .001* | .568-.868 |
| Physical Demand | 1.24 | .231 | 1.17 | .241 | .863-1.79 |
| Social Support | 1.11 | .132 | 0.90 | .366 | .882-1.40 |
| Noise | 1.42 | .215 | 2.35 | .019* | 1.06-1.91 |
| Trades Worker | .763 | .198 | -1.04 | .298 | .459-1.27 |
| Skilled Worker | .833 | .250 | -0.61 | .543 | .463-1.50 |
| Unskilled Worker | .798 | .239 | -0.75 | .452 | .444-1.44 |
| Marital Status | .928 | .032 | -2.17 | .030* | .867-.993 |
| Chinese | .482 | .238 | -1.48 | .139 | .183-1.27 |
| Sikh | 1.54 | .426 | 1.55 | .121 | .893-2.64 |
| Paternal Alcoholism | 1.80 | .599 | 1.77 | .076 | .939-3.46 |
| Paternal Mental Health | 1.12 | .142 | 0.85 | .393 | .868-1.43 |
| Paternal Suicidal Behaviours | 1.26 | .669 | 0.44 | .659 | .448-3.56 |
| *Adjustment Reaction* |  |  |  |  |  |
| Duration of Employment | .994 | .016 | -0.34 | .731 | .963-1.03 |
| Control | .980 | .038 | -0.51 | .613 | .908-1.06 |
| Psychological Demand | .960 | .107 | -0.37 | .711 | .771-1.19 |
| Physical Demand | .917 | .205 | -0.39 | .698 | .591-1.42 |
| Social Support | 1.25 | .185 | 1.50 | .133 | .934-1.67 |
| Noise | 1.02 | .186 | 0.09 | .928 | .710-1.46 |
| Trades Worker | 1.23 | .402 | 0.65 | .518 | .652-2.34 |
| Skilled Worker | 2.02 | .759 | 1.86 | .063 | .963-4.22 |
| Unskilled Worker | 1.50 | .571 | 1.07 | .286 | .712-3.16 |
| Marital Status | 1.02 | .034 | 0.53 | .593 | .953-1.09 |
| Chinese | .460 | .358 | -1.00 | .318 | .100-2.11 |
| Sikh | 1.52 | .476 | 1.33 | .185 | .820-2.81 |
| Paternal Alcoholism | .599 | .199 | -1.54 | .123 | .313-1.15 |
| Paternal Mental Health | .634 | .107 | -2.71 | .007* | .456-.882 |
| Paternal Suicidal Behaviours | 3.08 | 1.89 | 1.83 | .068 | .921-10.28 |
| *Depression* |  |  |  |  |  |
| Duration of Employment | 1.00 | .009 | 0.01 | .993 | .983-1.02 |
| Control | 1.01 | .021 | 0.25 | .800 | .966-1.05 |
| Psychological Demand | .882 | .058 | -1.91 | .057 | .775-1.00 |
| Physical Demand | 1.15 | .135 | 1.16 | .247 | .910-1.44 |
| Social Support | .971 | .074 | -0.38 | .702 | .837-1.13 |
| Noise | 1.01 | .094 | 0.11 | .914 | .842-1.21 |
| Trades Worker | .931 | .148 | -0.45 | .652 | .681-1.27 |
| Skilled Worker | .857 | .158 | -0.83 | .404 | .597-1.23 |
| Unskilled Worker | .847 | .155 | -0.90 | .366 | .592-1.21 |
| Marital Status | .955 | .020 | -2.18 | .029* | .916-.995 |
| Chinese | .617 | .208 | -1.43 | .152 | .319-1.19 |
| Sikh | .953 | .184 | -0.25 | .802 | .652-1.39 |
| Paternal Alcoholism | 2.06 | .491 | 3.03 | .002* | 1.29-3.29 |
| Paternal Mental Health | 1.19 | .096 | 2.16 | .031* | 1.02-1.39 |
| Paternal Suicidal Behaviours | .858 | .380 | -0.35 | .729 | .360-2.04 |
| *Alcohol Dependence* |  |  |  |  |  |
| Duration of Employment | .931 | .025 | -2.63 | .008* | .883-.982 |
| Control | .988 | .061 | -0.20 | .841 | .876-1.11 |
| Psychological Demand | .999 | .187 | -0.00 | .998 | .692-1.44 |
| Physical Demand | 1.45 | .559 | 0.97 | .334 | .682-3.09 |
| Social Support | .871 | .197 | -0.61 | .540 | .559-1.36 |
| Noise | 1.24 | .371 | 0.72 | .474 | .689-2.23 |
| Trades Worker | .896 | .538 | -0.18 | .855 | .276-2.91 |
| Skilled Worker | .697 | .453 | -0.56 | .578 | .195-2.49 |
| Unskilled Worker | .648 | .432 | -0.65 | .516 | .176-2.39 |
| Marital Status | .943 | .063 | -0.88 | .379 | .827-1.07 |
| Chinese | 2.09e-15 | 3.58e-8 | -0.00 | 1.00 | 0 |
| Sikh | .673 | .430 | -0.62 | .535 | .192-2.35 |
| Paternal Alcoholism | .495 | .249 | -1.40 | .162 | .185-1.33 |
| Paternal Mental Health | .896 | .252 | -0.39 | .697 | .517-1.56 |
| Paternal Suicidal Behaviours | 1.43 | 1.35 | 0.37 | .708 | .223-9.13 |
| *Drug Dependence* |  |  |  |  |  |
| Duration of Employment | .984 | .023 | -0.69 | .490 | .939-1.03 |
| Control | 1.03 | .058 | 0.51 | .612 | .922-1.15 |
| Psychological Demand | .804 | .134 | -1.31 | .190 | .580-1.11 |
| Physical Demand | 2.33 | .763 | 2.59 | .009* | 1.23-4.43 |
| Social Support | 1.17 | .224 | 0.80 | .423 | .800-1.70 |
| Noise | 1.21 | .291 | 0.80 | .426 | .756-1.94 |
| Trades Worker | .967 | .439 | -0.07 | .940 | .397-2.35 |
| Skilled Worker | .726 | .376 | -0.62 | .536 | .263-2.00 |
| Unskilled Worker | .716 | .377 | -0.63 | .526 | .255-2.01 |
| Marital Status | 1.01 | .047 | 0.27 | .784 | .924-1.11 |
| Chinese | 2.39e-15 | 2.20e-8 | -0.00 | 1.00 | 0 |
| Sikh | .729 | .431 | -0.53 | .593 | .229-2.32 |
| Paternal Alcoholism | .351 | .199 | -1.85 | .064 | .116-1.06 |
| Paternal Mental Health | .848 | .194 | -0.72 | .470 | .542-1.33 |
| Paternal Suicidal Behaviours | .438 | .483 | -0.75 | .454 | .050-3.80 |
| *Non-Dependent Drug Abuse* |  |  |  |  |  |
| Duration of Employment | .924 | .027 | -2.68 | .007* | .871-.979 |
| Control | .884 | .060 | -1.83 | .067 | .774-1.01 |
| Psychological Demand | 1.28 | .256 | 1.24 | .214 | .866-1.90 |
| Physical Demand | .720 | .294 | -0.80 | .422 | .323-1.61 |
| Social Support | 1.95 | .557 | 2.35 | .019* | 1.12-3.42 |
| Noise | 1.22 | .367 | 0.66 | .511 | .675-2.20 |
| Trades Worker | 1.72 | .901 | 1.04 | .301 | .616-4.80 |
| Skilled Worker | 1.31 | .772 | 0.45 | .650 | .411-4.16 |
| Unskilled Worker | .714 | .427 | -0.56 | .572 | .221-2.30 |
| Marital Status | .934 | .054 | -1.18 | .239 | .833-1.05 |
| Chinese | 2.47e-15 | 3.80e-8 | -0.00 | 1.00 | 0 |
| Sikh | 1.14 | .704 | 0.21 | .834 | .338-3.83 |
| Paternal Alcoholism | 4.36 | 2.82 | 2.28 | .023* | 1.23-15.5 |
| Paternal Mental Health | 2.15 | .546 | 3.02 | .003* | 1.31-3.54 |
| Paternal Suicidal Behaviours | 1.76 | 1.53 | 0.65 | .515 | .320-9.71 |

*p < .05
